# An Early Warning Risk Prediction Tool (RECAP-V1) for Patients Diagnosed With COVID-19: Protocol for a Statistical Analysis Plan

**DOI:** 10.2196/30083

**Published:** 2021-10-05

**Authors:** Francesca Fiorentino, Denys Prociuk, Ana Belen Espinosa Gonzalez, Ana Luisa Neves, Laiba Husain, Sonny Christian Ramtale, Emma Mi, Ella Mi, Jack Macartney, Sneha N Anand, Julian Sherlock, Kavitha Saravanakumar, Erik Mayer, Simon de Lusignan, Trisha Greenhalgh, Brendan C Delaney

**Affiliations:** 1 Department of Surgery and Cancer Imperial College London London United Kingdom; 2 Imperial Clinical Trials Unit Imperial College London London United Kingdom; 3 Patient Safety Translational Research Centre Institute of Global Health Innovation Imperial College London London United Kingdom; 4 Nuffield Department of Primary Care Health Sciences University of Oxford Oxford United Kingdom; 5 Whole Systems Integrated Care North West London Collaboration of Clinical Commissioning Group London United Kingdom; 6 Royal College of General Practitioners Research and Surveillance Centre London United Kingdom

**Keywords:** COVID-19, modeling, remote assessment, risk score, early warning

## Abstract

**Background:**

Since the start of the COVID-19 pandemic, efforts have been made to develop early warning risk scores to help clinicians decide which patient is likely to deteriorate and require hospitalization. The RECAP (Remote COVID-19 Assessment in Primary Care) study investigates the predictive risk of hospitalization, deterioration, and death of patients with confirmed COVID-19, based on a set of parameters chosen through a Delphi process performed by clinicians. We aim to use rich data collected remotely through the use of electronic data templates integrated in the electronic health systems of several general practices across the United Kingdom to construct accurate predictive models. The models will be based on preexisting conditions and monitoring data of a patient’s clinical parameters (eg, blood oxygen saturation) to make reliable predictions as to the patient’s risk of hospital admission, deterioration, and death.

**Objective:**

This statistical analysis plan outlines the statistical methods to build the prediction model to be used in the prioritization of patients in the primary care setting. The statistical analysis plan for the RECAP study includes the development and validation of the RECAP-V1 prediction model as a primary outcome. This prediction model will be adapted as a three-category risk score split into red (high risk), amber (medium risk), and green (low risk) for any patient with suspected COVID-19. The model will predict the risk of deterioration and hospitalization.

**Methods:**

After the data have been collected, we will assess the degree of missingness and use a combination of traditional data imputation using multiple imputation by chained equations, as well as more novel machine-learning approaches to impute the missing data for the final analysis. For predictive model development, we will use multiple logistic regression analyses to construct the model. We aim to recruit a minimum of 1317 patients for model development and validation. We will then externally validate the model on an independent dataset of 1400 patients. The model will also be applied for multiple different datasets to assess both its performance in different patient groups and its applicability for different methods of data collection.

**Results:**

As of May 10, 2021, we have recruited 3732 patients. A further 2088 patients have been recruited through the National Health Service Clinical Assessment Service, and approximately 5000 patients have been recruited through the DoctalyHealth platform.

**Conclusions:**

The methodology for the development of the RECAP-V1 prediction model as well as the risk score will provide clinicians with a statistically robust tool to help prioritize COVID-19 patients.

**Trial Registration:**

ClinicalTrials.gov NCT04435041; https://clinicaltrials.gov/ct2/show/NCT04435041

**International Registered Report Identifier (IRRID):**

DERR1-10.2196/30083

## Introduction

### Trial Background and Rationale

Since the start of the pandemic, there has been extensive work [1-3] to develop risk scores for the management of patients with acute COVID-19 that can help to predict the risk of hospitalization, deterioration, and death.

There is pressure on clinical services, and evidence that a small percentage of patients experience precipitous deterioration (usually on about day 7 after symptom onset) [4]. For this reason, there is a growing clinical need to develop and validate an early warning risk tool to be used in a primary care setting that is specific to COVID-19 and based on data that can be reliably collected during a remote consultation.

The RECAP (Remote COVID-19 Assessment in Primary Care) trial (NCT04435041) was designed to develop an early warning tool for patients diagnosed with COVID-19 in primary care settings. The original study protocol [5] outlines the study rationale and data collection process. This paper describes the process for quantitative development and validation of the RECAP-V1 model. The primary objective is to produce a multivariable risk prediction tool to facilitate primary care physicians and other clinicians working in the community in the early identification of COVID-19 patients that are at higher risk of requiring hospital admission, and to inform the early escalation of their treatment, with the aim to expedite admission where necessary and decrease deaths.

To minimize the risk of data selection bias and data-driven interpretation of results, we here outline a prespecified statistical analysis plan (Version 1.0), which details all of the analysis steps to develop and validate the risk prediction model.

### Research Questions

We aim to answer the following research questions: (1) What is the optimum risk model for RECAP-V1 to predict hospital admission? (2) What are the sensitivity, specificity, and positive and negative predictive values of the RECAP-V1 risk tool as used in the primary care assessment of COVID-19 patients?

### Study Objectives

The primary objective of the study is the development of a data-driven risk tool (RECAP-V1) for use in general practitioner (GP)-patient consultations (mainly by phone or video) in the context of COVID-19. As a secondary objective, the early warning risk tool will be externally validated on an independent dataset. As a third objective, the performance of the model in additional datasets will be assessed. Data for both model development and model validation will be prospectively collected.

## Methods

### Design

RECAP is a prospective cohort observational study. Data are remotely collected through a questionnaire form outlined in the original study protocol [5] for study-specific data, with additional demographic data being collected through routine electronic health record systems run by participating clinical practices.

### Sample Size

#### Model Development and Internal Validation

Assuming that 10% of patients diagnosed with COVID-19 will be admitted to hospital [6], a 0.05 acceptable difference in apparent and adjusted Cox-Snell R-squared, 0.05 margin of error in estimation of the intercept, a binary outcome based on admission to hospital, and a maximum of 24 predictor parameters [5], we estimate that the minimum sample size required for new model development is 1317 participants enrolled for the development set [7].

#### External Validation

For assessment of the prediction accuracy of the model, the sample size was calculated based on the assumption that 85% specificity would be the lowest level worth carrying forward because lower values would be considered too low for such a model to be used to make clinical decisions. We focus on specificity, because we are keen for the model to correctly identify the true negatives (and reduce the number of false negatives) considering the risk associated with missing a diagnosis. Based on a 95% CI and precision of 0.05, we aim to recruit at least 1400 patients for model external validation, assuming 87% specificity and a hospitalization rate of 10%.

Assuming a loss to follow-up of 5%-6%, due to possible linkage failure or not recording admission, we aim to recruit at least 2880 participants.

### Study Population

The main cohort will include patients presenting with clinically diagnosed COVID-19 in primary care requiring assessment of risk of clinical deterioration.

### Eligibility Criteria and Consent

Patients 18 years old and older, being seen (any form of contact, including face-to-face or remote) in a primary care setting where COVID-19 cases are occurring and running either a practice-based triage system or a COVID-19 remote monitoring service such as National Health Service (NHS)111 COVID-19 Clinical Assessment Service (CCAS) or a local equivalent, are enrolled in the study.

Patients being seen in practices not using a compatible electronic record system or using a remote monitoring system that cannot provide an output that is at least mapped to the appropriate Systemized Nomenclature of Medicine (SNOMED) concepts are excluded.

Patients locally record as being willing and able to provide informed consent for data linkage either at a GP contact (entered on a template) or as part of a “platform service” (checked by the patient on a template or via chatbot). If consent to data linkage cannot be obtained, an opt out will be provided and linkage sought under the Control of Patient Information provisions [8].

### Population Data

The data are collected in four different systems: (1) iCare (North West London Whole Systems Integrated Care), which is the Imperial analytics platform for high-paced processing of patient data from North West London practices; (2) the Royal College of General Practitioners (RCGP) Research and Surveillance network (RSC Practice network), which is a general practice sentinel network that collects data from practices across England and Wales; (3) NHS111 CCAS; and (4) DoctalyHealth Care, a home monitoring service for patients with a diagnosis of COVID-19.

The data will be collected using templates embedded in health record systems used in routine contacts for patients with suspected COVID-19, and then the records will be linked between primary and secondary care. The consent for this data linkage will also be collected. Once recruited, patients will be followed up for 28 days after the COVID-19 diagnosis.

### Definition of Analysis Population

The primary population set is patients presenting with clinically diagnosed COVID-19 in general practices and requiring assessment for the risk of clinical deterioration. The primary RECAP-V1 model will be built using data from iCare and externally validated using data from the RSC Practice network.

Additional population sets are patients that are part of DoctalyHealth, as well as patients who are being assessed by the NHS111 CCAS. Based on clinical expertise, the patients who are being assessed as part of NHS111 CCAS are expected to be experiencing more severe symptoms of COVID-19. Similarly, for DoctalyHealth, the data are collected from a home monitoring system; hence, we are expecting this patient population to be different from the iCare and RSC Practice network populations. Moreover, some of the important clinical parameters such as oxygen saturation or rate of breathing may be missing for DoctalyHealth.

A separate RECAP model will be built for the CCAS patients and the DoctalyHealth patients.

### Model Outcome Variable

The outcome variable considered in building the risk model RECAP-V1 is hospitalization within 28 days following a positive ascertained diagnosis of COVID-19 (either a clinical diagnosis or a polymerase chain reaction test).

Hospitalization is defined as a patient being admitted and having spent at least one night in a bed in a hospital in the period following a COVID-19 diagnosis and up to 28 days following the diagnosis.

### Model Predictor Variables

**Figure 1:**
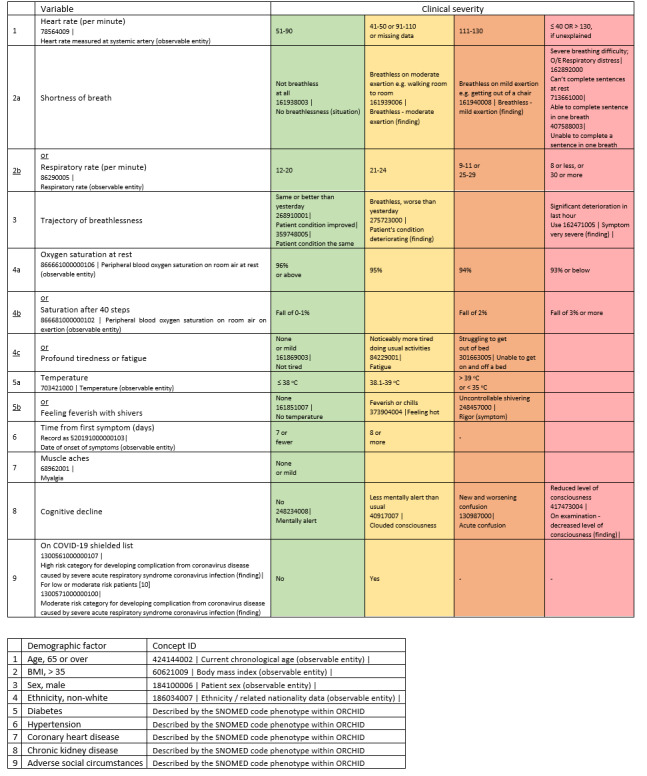
RECAP-V1 model predictor variables and their clinical severity, adapted from Greenhalgh et al [11]. O/E: on examination; ORCHID: Oxford-Royal College of General Practitioners Clinical Informatics Digital Hub; RECAP: Remote COVID-19 Assessment in Primary Care; SNOMED: Systemized Nomenclature of Medicine.

The predictor variables to be included as candidates for the model have been decided through a Delphi process by the investigators and the research team, which are presented in Figure 1. The SNOMED codes are also outlined for these predictors. These predictor variables are contained in RECAP V0 [9], which include patients’ sociodemographic information (eg, age, ethnicity, adverse social circumstances) and comorbidities. The predictors that will be included in the primary model are continuous (heart rate, respiratory rate, trajectory of breathlessness, oxygen saturation at rest, oxygen saturation after 40 steps, temperature, time from first symptom [days], age, BMI) and categorical variables (profound tiredness or fatigue, muscle aches, myalgia, cognitive decline, being on a COVID-19 shielded list [10], gender, ethnicity, diabetes, hypertension, coronary heart disease, chronic kidney disease, and adverse social circumstance).

### Statistical Methods

#### Baseline Demographics

Patient characteristics will be summarized. Summaries of continuous variables will be presented as means (SD) if normally distributed and as medians (IQR) for skewed data; categorical variables will be presented as frequencies and percentages. Baseline demographics will include all of the RECAP V0 variables outlined in Figure 1.

#### Objective 1 (Primary Objective): Development, Internal Validation, and Identification of Clinical Cut-Off Points of the RECAP-V1 Model

The primary objective of the study is to develop a predictive model based on a logistic regression of the predictive parameters using data from the iCare system. The model will be internally validated by bootstrapping the dataset. Following the internal validation, two cut-off points will be used as separators for red, amber, and green risk of a particular outcome with associated interval likelihood ratios (LRs).

A probabilistic risk prediction based on a multivariable logistic regression model, including the variables in Figure 1 as factors, will be performed on the patients from the iCare dataset, with continuous predictors being treated as fractional polynomials to reflect the potential nonlinearity of the variables. The model will allow estimation of the likelihood of a particular patient being admitted to hospital within 28 days of a COVID-19 diagnosis (primary patient outcome). Some of the variables will be checked for independence from each other by including interaction terms in the model. We will test interactions between BMI and respiratory rate (or shortness of breath if respiratory rate is not collected), age and respiratory rate (or shortness of breath), and coronary heart disease and respiratory rate (or shortness of breath).

The significance of each factor in the list of predictor parameters from Figure 1 will be investigated using a backward elimination regression model, where a multivariable regression model is constructed in the first instance including all of the parameters from the list in Figure 1 and their performance assessed to check for significance. Subsequently, a model using only the predictor factors that were found to be statistically significant (*P*<.05) for the prediction of results will be run.

**Figure 2:**
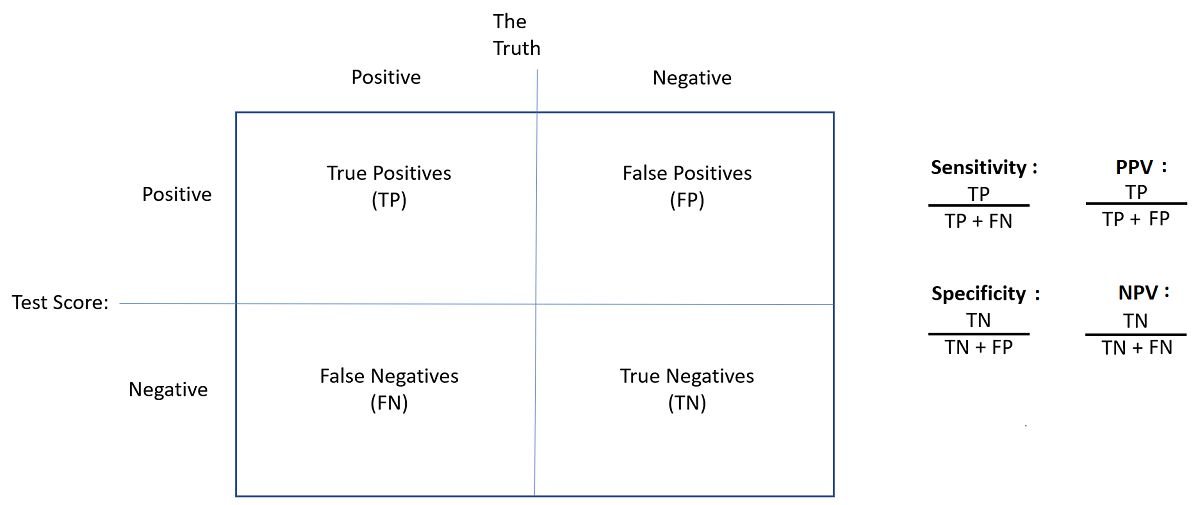
Sensitivity and specificity calculations based on test risk prediction and the real outcome, along with the positive and negative predictive value formulas. NPV: Negative Predictive Value; PPV: Positive Predictive Value.

The final model will then be used to estimate the predicted risk for each patient in the iCare dataset. Based on this continuous risk, the specificity and the sensitivity will be calculated based on the formulas shown in Figure 2. All analyses will be completed after the last patient’s final follow-up.

#### Model Internal Validation and Evaluation

The model will be validated internally by bootstrapping to ensure it performs well. The goodness of fit of the model will be assessed using the area under the receiver operating characteristic (ROC) curve and optimism-corrected smoothed calibration curves [12] to determine the performance of the model in predicting outcomes in the bootstrapped cohorts, with the final relationship between the predicted and the observed data being expressed as the McFadden R^2^ value [13].

To provide better representation of model fit, calibration and discrimination will be performed to better evaluate the accuracy of the model. Calibration of the model will be achieved using Loess-smoothed calibration plots as described above. For the purpose of analyzing discrimination, the data will be stratified by age (65 years old as a cut-off point) and gender. Hence, the model will be tested in population subgroups with age and gender for the subgroup definition. Brier scores will be used to measure the accuracy of the predictions of the model [14].

For each patient in this set, the result of the RECAP-V1 model will be calculated, and an ROC curve will be plotted from the highest to lowest values to determine the cut-off points for the predicted risk that optimize the specificity and sensitivity of the predictor model for red, amber, and green risk groups. The risk groups represent the relative risk of a particular patient outcome, with green signifying a very low risk, amber being a moderate to high risk, and red being a very high risk. This stratification ensures that there is a minimal number of false negatives in the final model and patients are not considered to be at low risk unless absolutely certain. The rate of increase and the point of inflection for the ROC curve will be used to inform the two cut-off points at the green/amber and amber/red points, which will be used as thresholds. The choice of optimum cut-off points is a clinical decision based on the shape of the resulting ROC curve (eg, how well-behaved the curve is, whether it is asymmetrical). Cut-off points should be clinically informative (ie, have a gradient representing the interval LR that is either greater than 2 or less than 0.5 so that the score changes the prior probability sufficiently). With a two cut-off point score for the red/amber/green (admit/monitor/advise) categories, the red/amber cut-off point should be in the steepest part of the curve (the most abnormal scores) where the LR will increase the risk of admission, and the amber/green cut-off point should be in the shallow part of the curve (the least abnormal scores) where the LR will decrease risk of admission. The ROC curve will thus be divided into three parts from the most abnormal to the least abnormal with three associated interval LRs; we will aim to allocate scores based on maximizing the “most abnormal” interval LR and minimizing the “least abnormal” interval LR with the middle interval LR likely to be around 1. There are several statistical tools for finding these points; however, in our opinion, selecting appropriate points from inspection of the ROC has the highest clinical validity [15].

#### Objective 2: External Validation of the Model

The model accuracy will then be assessed externally using the RSC Practice network data to verify the specificity of the model predictions, as well as the sensitivity, negative predictive value, and positive predictive value [16,17].

#### Objective 3: Analysis of Additional Datasets

A separate RECAP-V1 model will be developed, using the same methodology as described in Objective 1, for the CCAS and DoctalyHealth datasets, and the models will be internally validated following the same procedure as used in building the model for the iCare dataset.

#### Subgroup Analysis

The performance of the model will be investigated in specific groups of patients, focusing primarily on gender and age (<65 years).

#### Missing Data

The extent of missing data for each variable (outcome and predictors) will be assessed among all patients. Particular note will be taken of the potential for missing data in the oxygen saturation and temperature fields (in case the patient does not have instruments to measure these), and for respiratory rate (difficult to estimate unless independently counted visually) as these are considered particularly important factors for outcome.

If model outcome data are missing for >5% of the patients, methods to deal with missing data will be used and a sensitivity analysis to estimate the effect of using these methods will be carried out by comparing the estimated risk obtained without implementing a missing data method and the risk estimated using missing data methods.

The degree of missingness in predictor variables will be assessed. If the degree of missingness is above 50% for any predictor variable, then that predictor variable will be excluded from the model. If the degree of missingness is less than 50%, the data will be imputed using multiple imputation chain equations [18]. A total of 5 imputations will be performed and aggregated based on Rubin’s rules [19].

Data missingness mechanisms will be investigated to ascertain if the data are missing completely at random, at random, or not at random. This will be performed by considering the data structure to understand the dependency relationships between the missing data and the observed data. The missing data will then be described in relation to the degrees of missingness, missing data patterns, and possible reasons for missingness. We will also compare the characteristics of the patients with missing data and patients with complete data entries, which will allow us to assess the plausibility of the data missing completely at random. The distributions of the continuous predictor variables will be investigated for normality. Any missing continuous data that are not distributed normally will be transformed to a normal distribution for imputation and transformed back to the original scale for the final analysis.

Multiple imputation chain equations [18,20] will be used, and all of the predictor variables (with less than 50% missing data) will be included in the imputation model. Variables without missing data will also be included, and the outcome variable (hospital admission) will be used for imputation of the predictor variables. We will use linear regression for continuous variables (normally distributed or transformed), and logit or ordinal logit regression for categorical variables. We will compare observed and imputed values, especially for variables for which the fraction of missing data is large [21].

The same imputation methods will be used in all datasets, for both continuous and categorical variables.

#### Exploratory Analyses

We are planning to conduct exploratory analyses given the unique data collected in the study.

A time to event data model will be constructed using Cox regression of time to hospital admission. The model will be analyzed as time-series survival data by fitting a subdistribution hazard model [22] to account for deaths from any other causes that generate censored data in the results. This will be achieved by taking the time to admission for each patient. The discrimination of the models will be assessed using the Harrell C and Somer D statistics to measure the association of the ordinal logistic regressions.

A set of alternative risk estimates will also be explored in the possible scenarios for which temperature measurement, oxygen saturation at rest or on exercise, or respiratory rate are missing. It is expected that temperature, oxygen saturation, and respiratory rate might be at high risk of missingness, as in practice these factors might be hard to record. Hence, we will develop a prediction model in the case that these data are missing completely. This will also allow us to investigate the importance of these data for prediction of the risk of hospital admission.

A prediction model will be built using machine learning (ML). We will use nonlinear classifiers, including random forest and gradient tree boost algorithms, as well as a recurrent neural network to build a predictive model based on time-series data, as we expect there may be a nonlinear relationship between the predictors and the discrete outcomes. We will use 10-fold cross-validation with hyperparameter tuning by a grid search. RECAP-V1 built using logistic regression and the prediction models built using ML will be compared based on diagnostic accuracy estimates. ML methods for missing data imputation will also be applied. For this purpose, we will use random forest–based algorithms, DataWig, and generative models (generative adversarial networks), which have been shown to be superior to traditional missing data imputation techniques [23-25]. These ML methods will be compared to traditional missing data imputation.

Mortality and admission to the intensive care unit (ICU) will also be modeled. Two definitions of death from COVID-19 will be used: (1) death as a result of severe COVID-19 or COVID-19 complications in a hospital setting, and within 28 days of illness onset; and (2) death in a different setting (either after hospital discharge or in patients that were not admitted) within 28 days of illness onset, where COVID-19 is mentioned as a contributor in the death certificate of the patient. We will investigate the number of events for death and admission to the ICU, and determine if we have sufficient power to build predictive models for death and admission to the ICU.

Finally, we will explore combining the CCAS dataset to the primary iCare dataset to increase the power of the model. Differences in the patients between the datasets will be quantified, as well as exploring the effect that adding this additional dataset to the model would have on the performance of the model through internal-external cross-validation stratified for which dataset the patients’ data originated to ensure the transferability of the predictions for different subpopulations [16,26,27].

#### Software

Analyses will be performed using R studio 4.0 and STATA 15. The main R packages used for the analysis will be: rms, mice, miceMNAR, xplorerr, tidyverse, ggplot2, pubh, r2mlm, dplyr, tidyr, plotly, mlr3, and data.table.

## Results

A total of 173 active primary care practices have enrolled for the recruitment of patients across the iCare and RSC practice network. As of May 10, 2021, the study has recruited a combined sample of 3732 participants for the development, validation, and accuracy assessment of the model. A total of 2429 participants have been recruited from North West London GP practices (iCare), including the primary care data on the patients’ signs and symptoms during the full 28-day follow-up period; 1303 patients have been recruited from RSC practices, similarly recording the signs and symptoms of patients over the full 28-day follow-up period.

Through the DoctalyHealth platform, data have been collected using a remote monitoring system to record the patients’ clinical parameters, as well as signs and symptoms. The dataset comprises approximately 5000 patients and will be used to develop a model for this patient set. Additionally, 2088 patients have been recruited through NHS111 CCAS.

The final results of the model development and validation will be reported following the TRIPOD guideline [28] for prediction model development and validation. This study is expected to conclude in December 2021.

## Discussion

We have outlined the plan of analysis and methods for building a reliable data-driven early warning risk prediction tool, RECAP-V1, to be used in primary care.

COVID-19 has had a profound impact on the UK health care system, with limited numbers of ventilators and ICU beds. Thus, a method of early identification for not only the patients most at risk based on their demographic data, but also based on their disease symptoms and progression is of vital importance to efficiently treat the patients that need it the most. Previous early warning risk scores that were developed for the flu or generic infectious respiratory disease may not be entirely transferable to COVID-19, further highlighting the need for a reliable and data-driven risk prediction tool to help ensure the best outcomes for the highest risk groups.

Our RECAP-V1 early warning risk score will provide a robust, statistically supported metric for quickly assessing a patient’s current risk of hospitalization, and thus help clinicians decide if any change in treatment or closer observation would be warranted.

## Abbreviations

CCAS: COVID-19 Clinical Assessment Service
GP: general practitioner
ICU: intensive care unit
LR: likelihood ratio
ML: machine learning
NHS: National Health Service
RCGP: Royal College of General Practitioners
RECAP: Remote COVID-19 Assessment in Primary Care
ROC: receiver operating characteristic
SNOMED: Systemized Nomenclature of Medicine
